# Hepatitis C virus relapse after successful treatment with direct-acting antivirals, followed by sarcomatous changes in hepatocellular carcinoma: a case report

**DOI:** 10.1186/s13256-020-02392-y

**Published:** 2020-05-27

**Authors:** Ken Kurokawa, Takamasa Ohki, Jun Kato, Yukiyo Fukumura, Makoto Imai, Chikako Shibata, Junya Arai, Mayuko Kondo, Kaoru Takagi, Kentaro Kojima, Michiharu Seki, Masaya Mori, Nobuo Toda, Kazumi Tagawa

**Affiliations:** 1 Department of Gastroenterology, Mitsui Memorial Hospital, 1 Kandaizumi-cho Chiyoda-ku, Tokyo, 101-8643 Japan; 2 Department of Pathology, Mitsui Memorial Hospital, Tokyo, Japan

**Keywords:** Late viral relapse, Sarcomatous change, Sustained virologic response at 24 weeks (SVR 24)

## Abstract

**Background:**

Combination therapy of interferon and ribavirin has traditionally been used to eradicate hepatitis C virus. The sustained virologic response achieved with interferon-related therapy is persistent, and late relapses after achieving sustained virologic response at 24 weeks using this therapy are reportedly rare (< 1%). In 2014, interferon-free therapy with direct-acting antivirals was developed, and the rate of sustained virologic response was improved. However, the persistence thereof remains uncertain, and the appropriate follow-up period for hepatitis C virus-positive patients is under discussion.

**Case presentation:**

A 74-year-old Japanese man who had hepatitis C virus–related hepatocellular carcinoma and was successfully treated with radiofrequency ablation four times underwent direct-acting antiviral therapy with daclatasvir and asunaprevir; sustained virologic response at 24 weeks was confirmed. However, although he had no high risk factors for reinfection, hepatitis C virus ribonucleic acid was detected again 6 months after achieving sustained virologic response at 24 weeks. Moreover, he developed active hepatitis with an increased viral load. Five months after development of hepatitis, recurrent hepatocellular carcinoma emerged in segment II, where we had performed radiofrequency ablation 17 months previously. The recurrent hepatocellular carcinoma enlarged quite rapidly and induced multiple peritoneal disseminations and lung metastases. He died 3 months after the abrupt recurrence. A sarcomatous change in the hepatocellular carcinoma was identified during the autopsy.

**Conclusions:**

Although sustained virologic response at 24 weeks has generally been regarded to denote complete eradication of hepatitis C virus, we present a patient in whom hepatitis C virus recurred 6 months after achieving sustained virologic response at 24 weeks with direct-acting antiviral therapy. In addition, a sarcomatous change in hepatocellular carcinoma emerged 5 months after active hepatitis developed due to late hepatitis C virus relapse in this case. The sarcomatous change in hepatocellular carcinoma is generally thought to be related to anticancer therapies, such as radiofrequency ablation. However, in this case, late viral relapse and active hepatitis in addition to the previous radiofrequency ablation could have been the trigger. There may be a need for follow-up of hepatitis C virus ribonucleic acid beyond sustained virologic response at 24 weeks with direct-acting antiviral therapy, owing to the possibility of late viral relapse and tumorigenesis.

## Background

Chronic hepatitis C virus (HCV) infection induces a gradual progression to liver fibrosis, and the cumulative probability of developing hepatocellular carcinoma (HCC) is approximately 45% at 20 years after the initial infection [1]. Combination therapy of interferon (IFN) and ribavirin has traditionally been used to eradicate HCV. In 2014, IFN-free therapy with direct-acting antivirals (DAAs) was developed, and the rate of sustained virologic response (SVR) was improved. Although the SVR achieved with IFN-related therapy is persistent and late relapses after achieving sustained virologic response at 24 weeks (SVR 24) using this therapy are reportedly rare (< 1%) [2, 3], the frequency of late relapse after achieving SVR 24 with DAA therapy is still largely unknown. However, there have been several recent reports of late relapse in DAA-treated patients [4–6], and the appropriate follow-up period for HCV ribonucleic acid (RNA) positive patients after SVR is achieved remains controversial.

Although HCC is generally considered to be a slow-growing tumor, changes in tumor phenotypes, including speed of growth, occasionally occur. It has been reported that sarcomatous HCC, which is a subtype of HCC characterized by proliferation of spindle-shaped sarcomatoid carcinoma cells with unclear trabecular structures, can be induced by anticancer therapies, including radiofrequency ablation (RFA) and transcatheter arterial embolization (TAE) [7–9]. Sarcomatous HCC is likely to grow rapidly, invade extrahepatically, and result in a much poorer prognosis than ordinary HCC. However, triggers of sarcomatous changes, other than anticancer therapies, are unknown.

We present a case of a patient in whom HCV recurred at 6 months after achieving SVR 24 with DAA therapy, followed by sarcomatous changes in HCC. The possible relationship between the delayed HCV relapse and sarcomatous changes in HCC is discussed.

## Case presentation

Our patient was a 74-year-old Japanese man who had been diagnosed with chronic HCV at the age of 49 years in 1992. He did not achieve SVR either with IFN therapy in 1993 or with pegylated interferon (PEG-IFN)-α and ribavirin combination therapy in 2008. The first onset of HCC was in 2001, at which time it was treated with RFA. Segmentectomy of segment VI was performed in 2004 due to recurrence of the HCC. Thereafter, the patient underwent RFA in 2011, 2012, and 2014 for recurrent HCCs at segments VI, VIII, and VI, respectively.

**Fig. 1:**
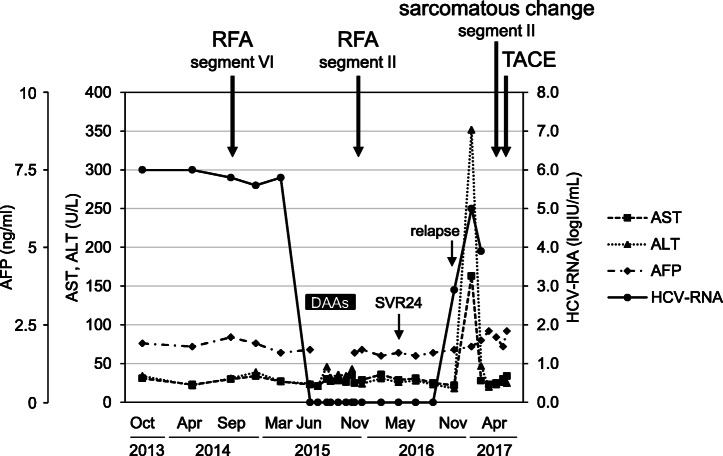
Clinical course of the patient. *DAA* Direct-acting antiviral, *HCV* Hepatitis C virus, *RFA* Radiofrequency ablation, *SVR 24* Sustained virologic response at 24 weeks, *TACE* Transcatheter arterial chemoembolization

In June 2015, the patient started DAA therapy with daclatasvir and asunaprevir, and SVR 24 was confirmed in May 2016 (Fig. 1). However, although he had no high risk factors for reinfection, HCV RNA (2.9 log IU/ml) was detected again in November 2016, 6 months after SVR 24. Moreover, he developed active hepatitis (aspartate transaminase 163 U/L [reference range 13–30 U/L] and alanine transaminase 352 U/L [reference range 10–42 U/L]), with an increase in the HCV RNA viral load (5.0 log IU/ml) seen in January 2017.

**Fig. 2:**
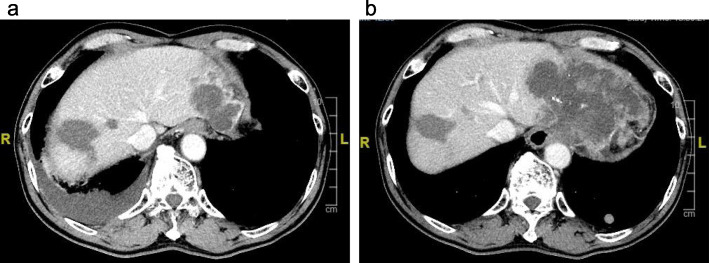
Contrast-enhanced computed tomography (CECT). CECT revealed multiple hepatocellular carcinoma (HCC) recurrences in the lateral segment (maximum size, ~ 4.0 × 3.0 cm) (**a**). The recurrent HCC enlarged quite rapidly (size 13.0 × 8.0 cm) and showed ringlike contour enhancement and central necrosis (**b**)

HCC recurrence occurred in segment II and was treated with RFA in November 2015, just after the patient finished the course of DAAs. After development of hepatitis due to relapse of HCV, multiple HCCs emerged in the lateral segment (maximum size ~ 4.0 × 3.0 cm) (Fig. 2a), and lung metastases were detected in April 2017. Transcatheter arterial chemoembolization using 70-mg miriplatin was performed, followed by oral sorafenib 400 mg/day.

He was admitted to our unit in June 2017 with abdominal pain due to a palpable mass in the upper abdomen. A contrast-enhanced computed tomographic scan showed that the recurrent HCC in the lateral segment had enlarged quite rapidly (13.0 × 8.0 cm), showing ringlike contour enhancement and central necrosis (Fig. 2b). Only palliative care was provided, and he died 3 months after the abrupt multiple recurrence.

**Fig. 3:**
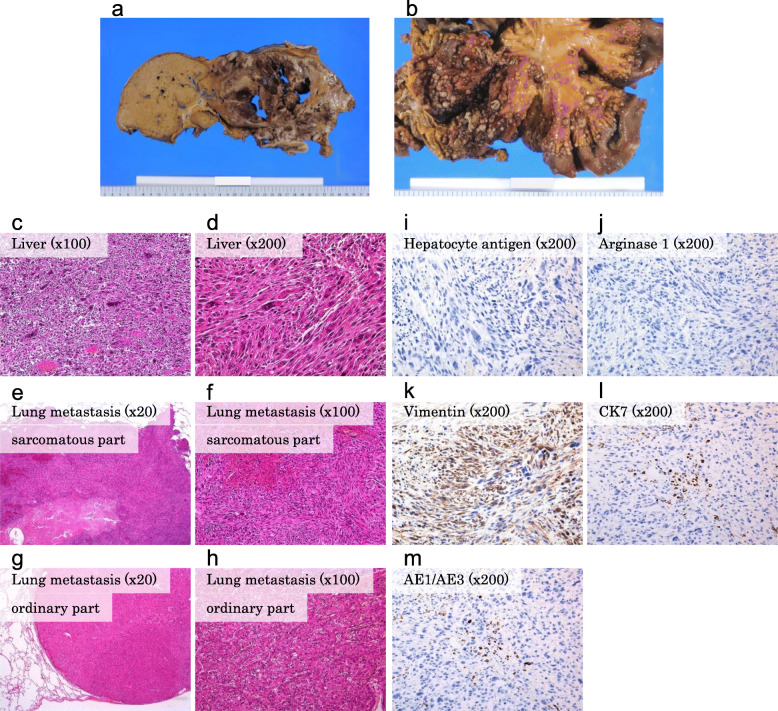
Pathological analysis. Autopsy revealed that the left lobe of the liver was filled with tumors, accompanied by central necrosis (**a**). The tumors exhibited extrahepatic growth, including invasion into the stomach and left diaphragm. Severe peritoneal dissemination was also detected (**b**). The pathologic tests demonstrated that the tumors consisted of multiple spindle-shaped cells, suggesting a sarcomatous change in hepatocellular carcinoma (HCC) (**c**, **d**). Both sarcomatous and ordinary HCCs were identified as lung metastases (**e**–**h**). The result of immunohistochemical staining of the spindle-shaped cells was negative for hepatocyte antigen and arginase 1 (**i**, **j**). In contrast, vimentin was positively stained (**k**), and cytokeratin 7 (CK7) and pankeratin (AE1/AE3) appeared to be slightly positive (**l**, **m**)

The autopsy of our patient revealed that the left lobe of the liver was filled with tumors, accompanied by central necrosis (Fig. 3a). The tumors showed extrahepatic growth, including invasion into the stomach and left diaphragm, and peritoneal and pleural dissemination (Fig. 3b). Multiple lung metastases were also detected. Pathologic tests demonstrated that the tumors consisted of multiple spindle-shaped cells, suggesting a sarcomatous change in HCC (Fig. 3c, d). Both sarcomatous and ordinary HCCs were observed as lung metastases (Fig. 3e–h). The result of immunohistochemical staining of the spindle-shaped cells was negative for hepatocyte antigen and arginase 1, both of which are common HCC markers (Fig. 3i, j). In contrast, the result for the mesenchymal marker vimentin was positive (Fig. 3k), and the results for epithelial markers, including cytokeratin 7 and pankeratin (AE1/AE3), appeared to be slightly positive (Fig. 3l, m). These findings suggested that the tumors originated from the sarcomatous change in HCC.

## Discussion and conclusions

Our patient’s case was characterized by two important clinical events: (1) redevelopment of HCV RNA at 6 months after achieving SVR 24 with DAA therapy and (2) a sarcomatous change in HCC.

During the era of IFN-related therapy, SVR at 12 weeks (SVR 12) was sufficient to demonstrate viral eradication because of the high concordance between SVR 12 and SVR 24 [10]. In addition, late relapse after achieving SVR 24 with IFN-related therapy is quite rare (< 1% of cases) [2, 3]. A high concordance between SVR 12 and SVR 24 has been reported in patients who underwent DAA therapy [11]. However, Sarrazin *et al.* reported that, of 3004 patients achieving SVR 12 with sofosbuvir-based DAA therapy, HCV RNA recurred before SVR 24 in 5 patients (0.2%) [12]. In a more recent study, HCV RNA was detected again in serum at 6, 12, 12, and 26 months after SVR 24 in 4 of 413 patients who completed DAA therapy (daclatasvir and asunaprevir) [6]. Thus, late relapse after successful DAA therapy remains controversial.

In our patient’s case, although DAA therapy (daclatasvir and asunaprevir) was completed and SVR 24 was confirmed, HCV RNA was detected again in the serum at 6 months after SVR 24, with subsequent development of hepatitis. Reinfection of HCV could not be completely ruled out because of the lack of HCV RNA sequencing data. However, the possibility seemed quite small, because the patient had no risk factors for reinfection (injected drug use or sexual activity). Although the mechanism of late relapse remains unclear, a small (undetectable) amount of HCV in the serum might remain in hepatocytes and/or peripheral blood mononuclear cells after SVR 24 with DAA therapy [13–15]. HCV is eradicated by upregulating the immune system via traditional IFN-related therapy, whereas DAA therapy directly eradicates HCV without activating the immune system; this may have influenced the late relapse in our patient’s case. Further follow-up beyond SVR 24 may be needed in HCV RNA-positive cases treated with DAA therapy.

A sarcomatous change in HCC also occurred in this case. After HCV recurrence, HCC recurred in segment II and grew quite rapidly. Immunohistochemical analysis during the autopsy revealed the sarcomatous change in HCC. The incidence of sarcomatous change in HCC has been increasing and is thought to be related to anticancer therapies, such as TAE, percutaneous ethanol injection therapy, and RFA [7–9]. Kojiro *et al.* showed that among 579 autopsy cases of HCC, 55 (9.4%) exhibited a sarcomatous change. In addition, the change was found in 20.9% of patients who underwent anticancer therapy, whereas it was detected in only 4.2% of patients without anticancer therapy [7]. Although RFA is one of the main treatments for focal HCC, HCC recurrence with sarcomatous change after RFA was also reported [9]. In fact, five cases showing sarcomatous changes, including our patient’s case, have been observed at autopsy at our institute (Table 1); all of them received anticancer therapy.

**Table 1:** Five cases showing sarcomatous changes in hepatocellular carcinoma

Case	Age (years)	Sex	Background	HCV Ab	AFP (ng/ml)	PIVKA-II (mAU/ml)	Central necrosis	Extrahepatic growth	Distant metastasis	Treatment before sarcomatous change
1	81	Male	Chronic hepatitis	+	n.a.	n.a.	–	+	+	PEIT
2	66	Male	Liver cirrhosis	+	756	39,700	+	+	+	TAE
3	74	Female	Liver cirrhosis	+	4.4	20	+	+	+	RFA
4	83	Female	Liver cirrhosis	+	5926	14	–	–	+	RFA, TACE
Our patient	74	Male	Liver cirrhosis	+	2.3	19	+	+	+	RFA

*Abbreviations: AFP* α-Fetoprotein, *n.a.* Not available, *PEIT* Percutaneous ethanol injection therapy, *PIVKA-II* Protein induced by vitamin K absence/antagonist-II, *RFA* Radiofrequency ablation, *TACE* Transcatheter arterial chemoembolization, *TAE* Transcatheter arterial embolization

Five cases with sarcomatous changes, including our patient’s case, have been observed at autopsy at our institute

Although the mechanism underlying sarcomatous HCC remains unclear, factors including anticancer therapy may change the phenotype of cancer cells or induce selection of a clone with a sarcomatous nature. Few authors have reported on the relationship between HCV antiviral therapy and sarcomatous changes in HCC. Idobe-Fujii *et al.* reported that sarcomatous changes in HCC emerge after achieving SVR with IFN-related therapy [16]. Authors of a recent study reported that rapidly growing, dedifferentiated HCC is more likely to emerge in patients who achieve SVR with DAA therapy [17]. Thus, antiviral therapy might effect a change in the HCC phenotype, but further observational studies are required to verify this. In our patient’s case, sarcomatous HCC emerged 5 months after active hepatitis developed due to late HCV relapse. The inflammation associated with active hepatitis may also have triggered the sarcomatous change in HCC.

Although SVR 24 has generally been regarded to denote complete eradication of HCV, we present a case of a patient in whom HCV recurred 6 months after achieving SVR 24 with DAA therapy; a sarcomatous change in HCC was also seen. This is the first report showing that late relapse of HCV might contribute to a sarcomatous change in HCC, in addition to previous anticancer therapies. Follow-up of HCV RNA beyond SVR 24 with DAA therapy may be needed due to the possibility of late relapse and tumorigenesis.

## Data Availability

The datasets used and analyzed during this study are available from the corresponding author on reasonable request.

## Abbreviations

AFP: α-Fetoprotein
DAA: Direct-acting antiviral
HCC: Hepatocellular carcinoma
HCV: Hepatitis C virus
IFN: Interferon
PEG-IFN: Pegylated interferon
PEIT: Percutaneous ethanol injection therapy
PIVKA-II: Protein induced by vitamin K absence/antagonist-II
RFA: Radiofrequency ablation
SVR: Sustained virologic response
SVR 12: Sustained virologic response at 12 weeks
SVR 24: Sustained virologic response at 24 weeks
SVR: Sustained virologic response
TACE: Transcatheter arterial chemoembolization
TAE: Transcatheter arterial embolization
